# Patient perspectives on SLE, refractory APS, and biologic drug use during pregnancy

**DOI:** 10.1177/09612033261437466

**Published:** 2026-03-26

**Authors:** Jara van Woerkom, Merlijn Wind, Yoe Kie Onno Teng, Titia Lely, Jamy Pullen, Gerard Jansen, Luis Perez-Garcia, Radboud Dolhain, Maarten Limper, Judith Kooiman

**Affiliations:** 1 Department of Obstetrics, 8124 University Medical Center Utrecht, Utrecht, The Netherland; 2 Department of Obstetrics, 4501 Leiden University Medical Center, Leiden, The Netherlands; 3 Department of Nephrology, 4501 Leiden University Medical Center, Leiden, The Netherlands; 4 Dutch National Patient Association for Lupus Erythematosus (NVLE), Utrecht, The Netherlands; 5 Department of Hematology, 6984 Erasmus University Medical Center, Rotterdam, The Netherlands; 6 Department of Rheumatology, 6984 Erasmus University Medical Center, Rotterdam, The Netherlands; 7 Department of Rheumatology and Clinical Immunology, 8124 University Medical Center Utrecht, Utrecht, The Netherlands; 8 Department of Obstetrics, 6984 Erasmus University Medical Center, Rotterdam, The Netherlands

**Keywords:** Pregnancy, antiphospholipid syndrome, systemic lupus erythematosus

## Abstract

**Purpose:**

Women with Systemic Lupus Erythematosus (SLE) and/or Antiphospholipid Syndrome (APS) have an increased risk of pregnancy complications. This study explored their perspectives on pregnancy, pregnancy complications, and medication use during pregnancy.

**Methods:**

In collaboration with the Dutch National Patient Association for Lupus Erythematosus, an online questionnaire was developed and distributed among female members. The questionnaire presented eight potential pregnancy complication scenarios. Respondents indicated whether they found the standard medication regime sufficient and their willingness to take additional medication. They also assessed which side effects of biological therapies would discourage additional treatment. The survey ran from November 18, 2023, to March 19, 2024.

**Major findings:**

Of 71 respondents (mean age 39), 42% had APS, 31% had SLE, and 27% had both. A majority (79%) would endure pregnancy complications, such as SLE flare or pre-eclampsia, if it meant a live-born baby. Willingness to use additional medication varied by scenario (51%–89%) depending on the specific complication at risk. Most side effects associated with the use of biologicals were deemed acceptable by the majority of respondents. However, a slight majority indicated they would refrain from additional medication if it led to an increased risk of infections such as urinary tract infections or pneumonia.

**Conclusion:**

Most women would accept adverse obstetric outcomes or maternal complications as long as pregnancy results in a live birth. The majority of respondents consistently expressed a desire for additional medication to reduce the risk of complications during pregnancy, regardless of the specific complication at hand.

## Introduction

Systemic Lupus Erythematosus (SLE) and Antiphospholipid Syndrome (APS) are rare autoimmune diseases that typically affect women of childbearing age.^1–3^ APS can exist as primary APS, obstetric APS or related to underlying autoimmune disease such as SLE.^2^ While infertility rates among women with these conditions are comparable to those in the general population,^4^ they face an increased risk of severe obstetric complications or disease flare during pregnancy.^2,3^

The European Alliance of Associations for Rheumatology and American College of Rheumatology guidelines advise treating patients with SLE with hydroxychloroquine throughout gestation, alongside other immunosuppressive agents that effectively maintain disease remission and are compatible with pregnancy, if needed.^1,5,6^ According to these same guidelines, patients with APS are advised to use aspirin and low-molecular-weight heparin during pregnancy, as this therapeutic approach has been shown to increase life-birth rates and to prevent thrombosis during pregnancy and the puerperium.^1,5,7–9^

However, despite these guideline recommended treatments, patients with SLE and/or APS still face elevated risks of adverse pregnancy outcomes compared to the general population.^1^ For example, patients with SLE are still at increased risk of preterm birth (incidences varying between 25- 35%), pre-eclampsia (10-15%), fetal growth restriction and disease flare.^1,9,10^ For primary APS, the risks of preterm birth and pre-eclampsia are even higher, respectively 25-35% and 10-20%. Additionally, 54% of women with obstetric APS have recurrent miscarriages despite current treatment.^11^

These high complication risks drive the scientific and clinical community to search for additional immunomodulating agents to improve pregnancy and maternal outcomes in patients with SLE and APS.^12^ It is however unclear whether patients also experience a need for additional therapy during pregnancy, and whether they would be willing and feel safe to use additional biological disease-modifying antirheumatic drugs for example. The aim of this study was to investigate the perspectives of SLE and/or APS patients regarding pregnancy, pregnancy complications, and medication use during pregnancy. Specifically, the study aimed to identify complications that patients believe should be avoided, determine the circumstances in which patients desire additional medication, and assess when the prevention of potential complications outweighs the risk of potential side effects related to the use of biologic drugs.

## Methods

### Survey

A literature search was conducted to identify potentially useful validated surveys exploring patients’ perspectives on medication use during pregnancy in individuals with APS or SLE (search string in Appendix I). None of the identified questionnaires were suitable for the aim of this study. For instance, The Beliefs about Medicine Questionnaire was not suitable for our research question as it is designed to estimate beliefs about medication in general, not specifically during pregnancy. Consequently, a new patient questionnaire was created in Dutch in collaboration with the spokeswoman of the patient association of the Dutch National Association of Lupus Erythematosus (NVLE). The questionnaire was first tested in a pilot phase involving 10 women, and their responses were reviewed. Subsequently, adjustments were made to the questions where necessary. The final version of the questionnaire, translated into English, can be found in Appendix II.

The online survey was created using Spidox software, an online tool specifically designed for patient questionnaires that complies with international privacy regulations. The questionnaire included a combination of open-ended and structured questions. Initially, patients were asked about their age, autoimmune disease diagnosis, desire to have children, and, if relevant, any previous pregnancies they had experienced.

Subsequently, open-ended questions were posed exploring the respondents perceptions on what constitutes a successful and unsuccessful pregnancy outcome. Additionally, the survey introduced three scenarios outlined in vignettes: mild pre-eclampsia, severe pre-eclampsia, and recurrent miscarriages. Background information, including the incidences of these complications in the general population and among patients with APS or SLE, was provided to the respondents. Participants were then asked to assess whether they perceived these scenarios as complications, whether they deemed the current line of treatment adequate, and if they would consider using additional medication to lower the risk of the specific complications outlined in the vignette.

Subsequently, the survey described eight potential complications, each accompanied by inquiries regarding the adequacy of medication use (sufficient or insufficient) and the consideration of additional medication to reduce the risk of the specific complication. The questionnaire concluded with an inquiry about which side effects would deter respondents from taking additional medication during pregnancy. Side effects listed were based on side effects associated with the use of biological disease modifying anti-rheumatic drugs.

The protocol of the study was approved by the Ethics Committee of the University Medical Center Utrecht (No. Ethics Code: 23U-0294).

### Study cohort

The Dutch National Association of Lupus Erythematosus (NVLE) requested its female members to fill out the questionnaire via an invitational email with a link to the questionnaire. This association comprises nearly 2500 members, including 1300 females with either APS, SLE, or both conditions. Patients with primary APS were eligible to complete the questionnaire, regardless of whether their APS was refractory. Prior to this invitation, an online webinar on pregnancy in APS and SLE was conducted for NVLE members to draw attention to the survey as well as serve as an educational initiative on the topic. Additionally, the questionnaire was shared on various social media platforms, associated with the NVLE (LinkedIn, Facebook, Instagram). The online questionnaire was accessible from November 18^th^, 2023 till March 19^th^, 2024. Respondents who did not report a diagnosis of APS or SLE were excluded from the study analysis.

### Data extraction and analysis

After the closure of the online questionnaire, the NVLE extracted outcomes from the online platform. Upon commencement of the analysis, it became evident that certain responses were duplicated as some answers to the open questions were exactly similar. To uphold data integrity, the following criteria were established to filter out any duplicate responses:

(i) Respondents of the same age;
(ii) A minimum of 90% similarity in answers to the fixed questions;
(iii) Answers to all open-ended questions must be exact or substantially similar, indicating identical essence across all three questions, while allowing for variations in punctuation and language usage.

Responses meeting all three criteria were marked as duplicates. When identified as duplicates, the most comprehensive response was utilized in the study analyses. The results were anonymized and summarized by the NVLE. The fixed question data were analysed descriptively, while the free text data were analysed using thematic context analysis. All comments were read, and key emergent themes were noted. The most frequently used words were visualised using online software, specifically the WordArt tool, to represent them in a word cloud. If respondents missed a question, it was marked as ‘missing’ in the results.

## Results

The online questionnaire received 102 responses. After excluding 12 incomplete submissions and 19 duplicates, 71 unique responses were available for analysis.

The median age of the respondents was 39 years, ranging from 19 to 73 years. Among the 71 respondents, 42% had been diagnosed with APS, 31% with SLE, and 27% was diagnosed with APS secondary to SLE.

A total of 89% of patients expressed a desire to have children. Among those who desired children, mean age was 32.6 years (range 19-44 years), 57% had already given birth to a live-born baby, while 40% did not. Two respondents did not provide an answer to this question.

Overall, 79% of the respondents stated that they would accept complications if it meant having a live-born baby. Specifically, 62% of patients were willing to accept pre-eclampsia and 25% to accept thrombosis if pregnancy would result in a live-born neonate. Among women diagnosed with SLE, 66% were willing to accept an SLE flare if pregnancy would result in a live birth Table 1.

**Table 1.** Patient characteristics according to age.

Age groups	< 30 (*n* = 13)	30-45 (*n* = 48)	> 45 (*n* = 10)
Diagnosis			
APS	0 (0)	30 (63)	0(0)
SLE	10 (77)	4 (8)	8 (80)
Both	3 (23)	14 (29)	2 (20)
Desire to have children			
Yes	12 (92)	44 (92)	7 (70)
No	1 (8)	4 (8)	3 (30)
Live born baby (% of women with desire for children)			
Yes	1 (8)	28 (64)	7 (100)
No	9 (75)	16 (36)	0 (0)
Missing	2 (17)	0 (0)	0 (0)

### Perspectives on complications and medication use in pregnancy: vignettes

**Figure 1.**
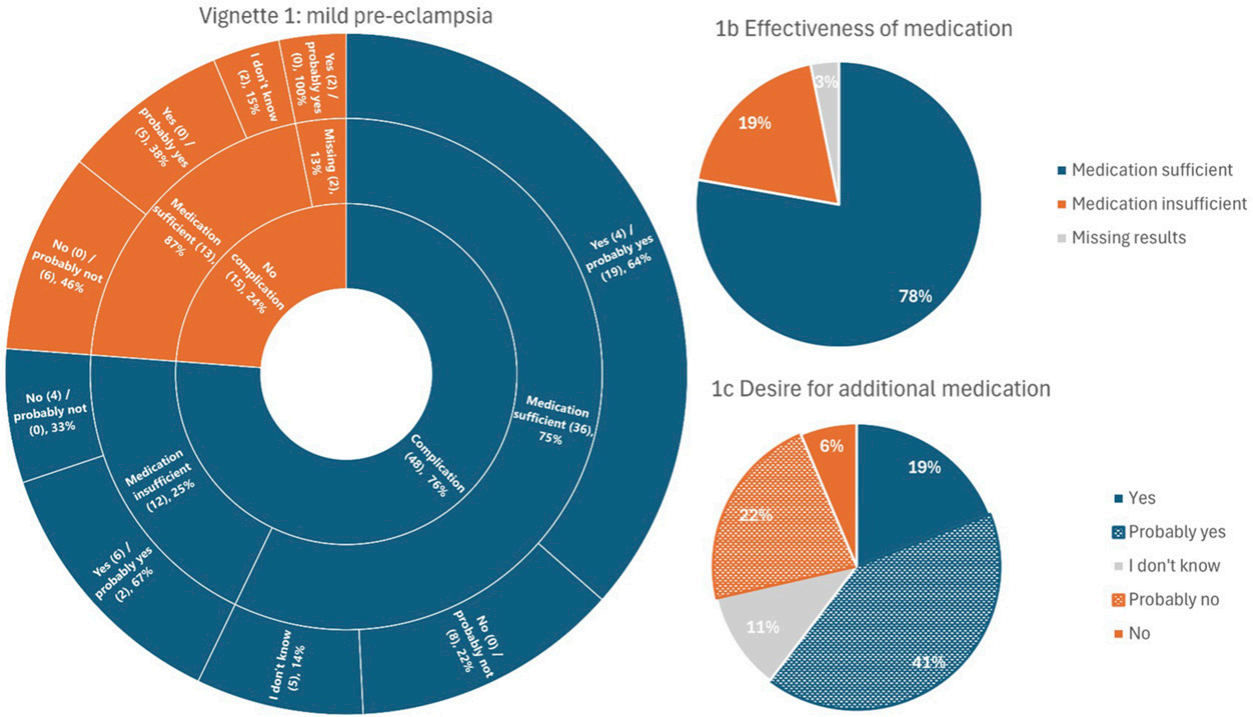
Vignette 1 – mild pre-eclampsia. A woman develops mild pre-eclampsia (high blood pressure and symptoms such as headaches, fluid retention, and pain in the upper abdomen) despite standard therapy. Her baby is born healthy around the due date. The figure is structured from the inner ring to the outer ring, representing the questions: ‘Do you consider this a complication?’, ‘Do you believe the medication used was working sufficiently?’, and ‘Would you consider taking additional medication in this situation?’. The numbers in parentheses (n) represent the absolute number of respondents who provided this answer. 1b represents the question ‘Do you believe the medication used was working sufficiently?’ for the whole group. 1c represents the question ‘Would you consider taking additional medication in this situation?’ for the whole group.

**Figure 2.**
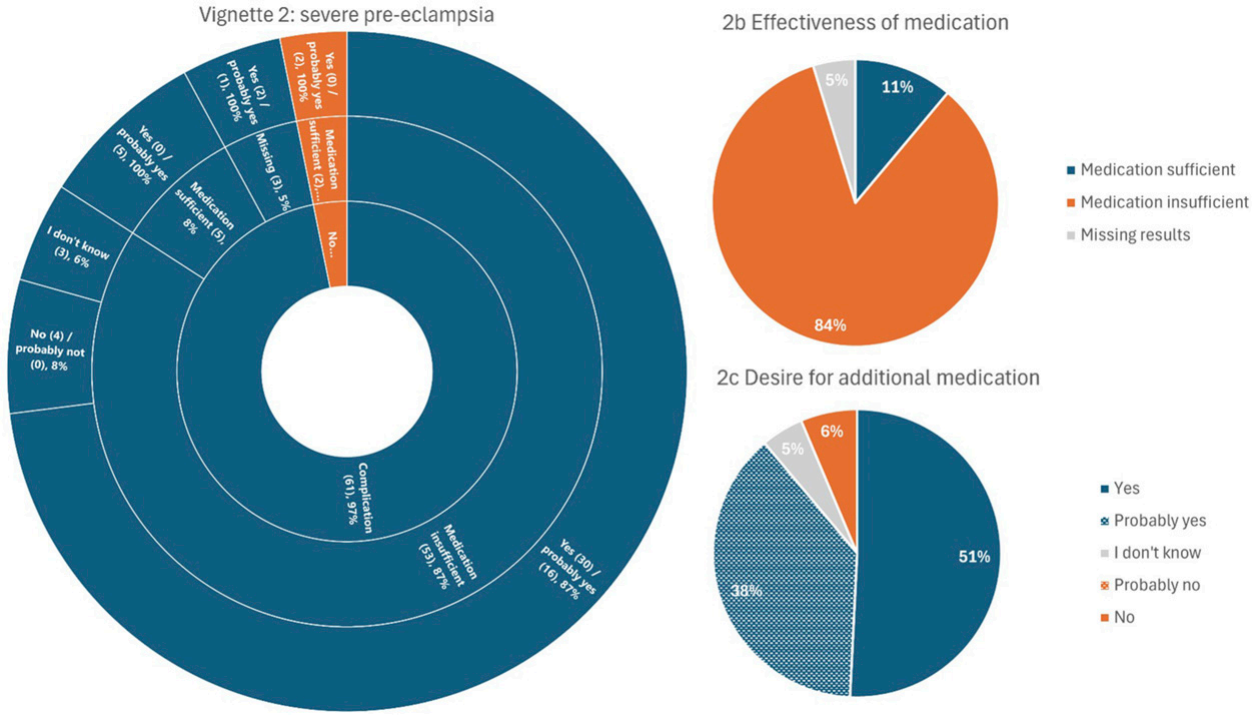
Vignette 2 – severe pre-eclampsia. A woman develops severe pre-eclampsia at 32 weeks of gestation, resulting in her baby being born prematurely. The figure is structured from the inner ring to the outer ring, representing the questions: ‘Do you consider this a complication?’, ‘Do you believe the medication used was working sufficiently?’, and ‘Would you consider taking additional medication in this situation?’. The numbers in parentheses (n) represent the absolute number of respondents who provided this answer. 2b represents the question ‘Do you believe the medication used was working sufficiently?’ for the whole group. 2c represents the question ‘Would you consider taking additional medication in this situation?’ for the whole group.

**Figure 3.**
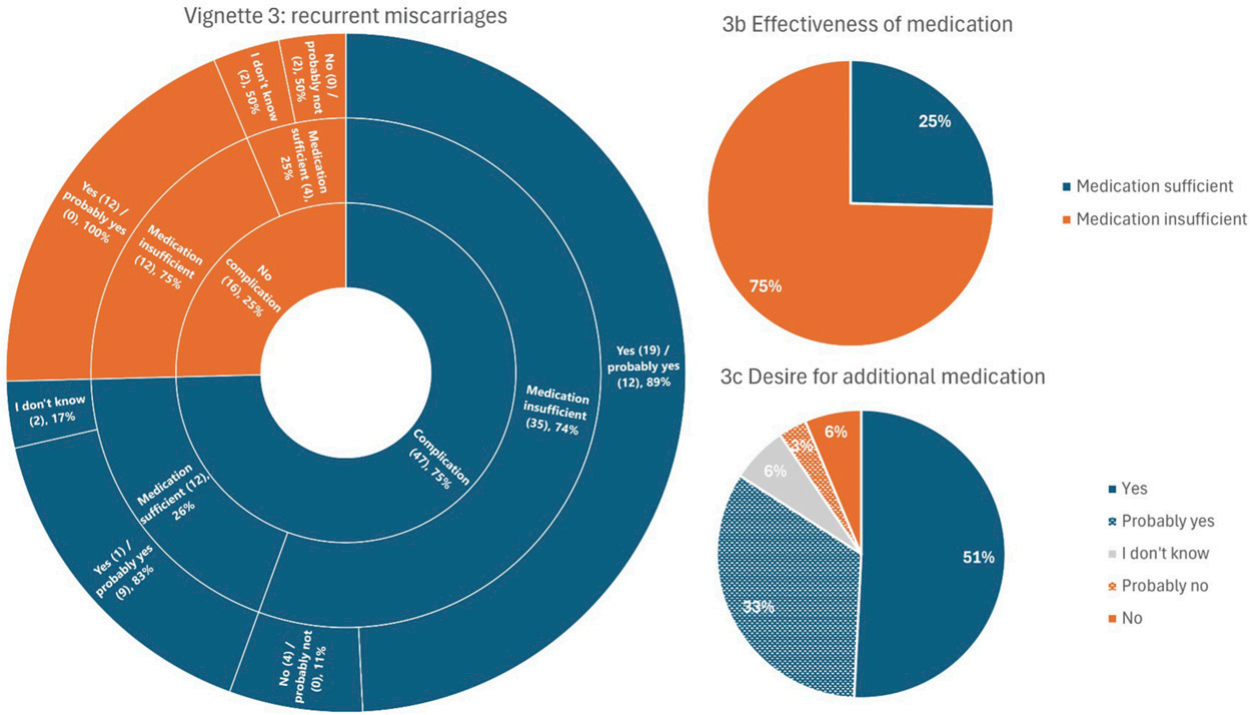
Vignette 3 – recurrent miscarriages. A woman has multiple miscarriages. After several years, she has a successful pregnancy and brings home a healthy baby. The figure is structured from the inner ring to the outer ring, representing the questions: ‘Do you consider this a complication?’, ‘Do you believe the medication used was working sufficiently?’, and ‘Would you consider taking additional medication in this situation?’. The numbers in parentheses (n) represent the absolute number of respondents who provided this answer. 3b represents the question ‘Do you believe the medication used was working sufficiently?’ for the whole group. 3c represents the question ‘Would you consider taking additional medication in this situation?’ for the whole group.

Figures 1-3 depict the responses to the vignettes described in the questionnaire. From the inner to the outer ring, it represents the following questions: ‘Do you consider this a complication?’, ‘Do you believe the medication used was effective enough?’, and ‘Would you consider taking additional medication to lower the risk of the complication described in this scenario?’.

#### Vignette 1: mild pre-eclampsia

##### Description

A woman Figure 1 develops mild pre-eclampsia (high blood pressure and symptoms such as headaches, fluid retention, and pain in the upper abdomen) despite standard therapy. Her baby is born healthy around the due date.

Among the respondents, 76% considered mild pre-eclampsia to be a complication of their rheumatic disease in pregnancy. Despite this, 75% of these respondents believed that the medication used was effective enough. Among respondents who did not consider mild pre-eclampsia a complication, a majority of 87% believed the medication used was effective. Nevertheless, 61% of all respondents expressed willingness to take additional medication to lower the risk of developing mild pre-eclampsia.

#### Vignette 2: severe pre-eclampsia

##### Description

A woman develops severe pre-eclampsia at 32 weeks of gestation, resulting in her baby being born prematurely Figure 2.

Severe pre-eclampsia was regarded as a complication by 97% of the respondents and 87% believed standard medication to be ineffective. In total, 89% of all respondents wanted additional medication to prevent severe pre-eclampsia, while 6% did not, and 5% was uncertain.

#### Vignette 3: recurrent pregnancy loss

##### Description

A woman has multiple miscarriages. After several years, she has a successful pregnancy and brings home a healthy baby Figure 3.

Recurrent pregnancy loss was considered a complication by 75% of respondents. Overall, 25% answered that the medication used was effective enough, while 75% thought treatment failed. In total, 84% of respondents expressed a desire for additional medication, with 10% opposed and 6% was unsure.

### Medication preferences across pregnancy complications and diagnoses

**Figure 4.**
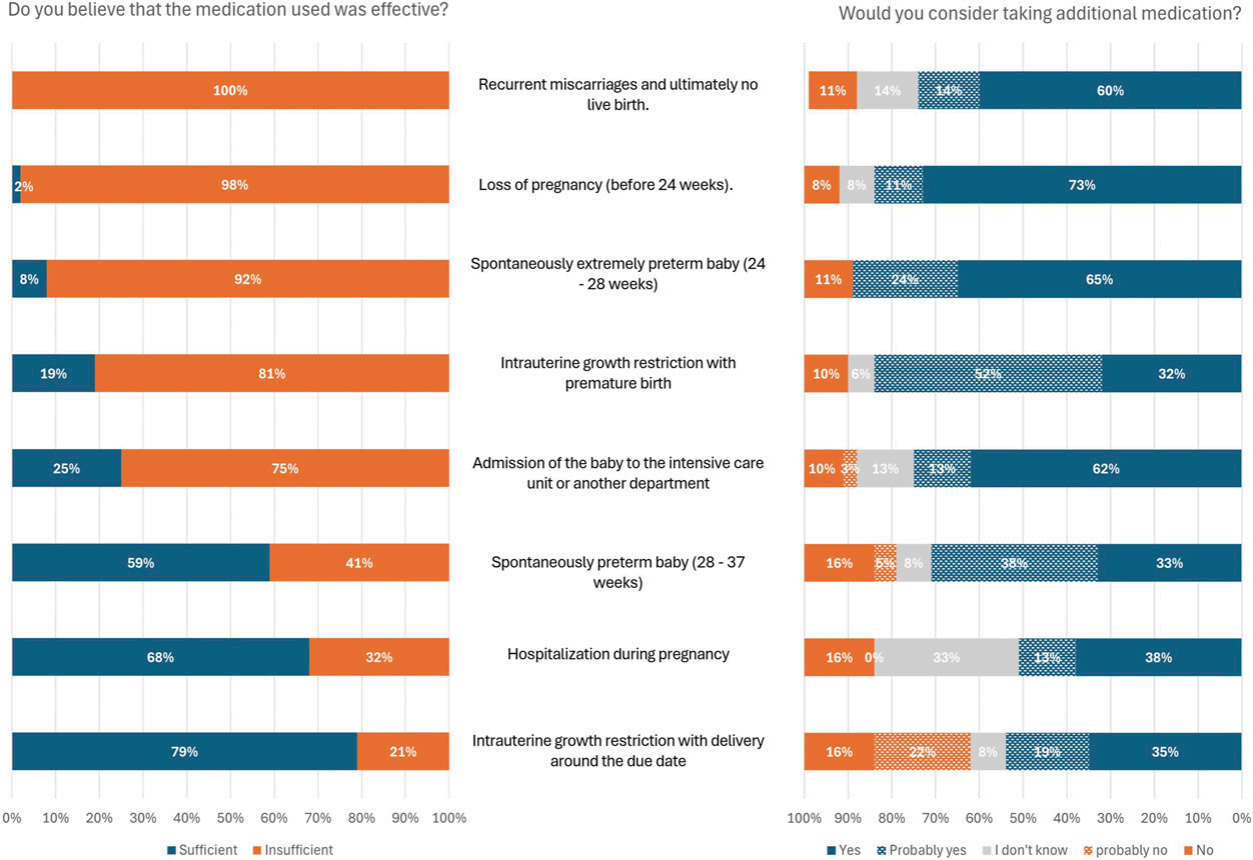
Eight scenarios were described in the middle, each accompanied by the following two questions: on the left, ‘Do you believe that the medication used was effective?’, and on the right ‘Would you consider taking additional medication in this situation?’.

Figure 4 presents the reflections of respondents on eight scenarios describing complications during pregnancy. For each scenario, patients were asked whether they believed the standard medication regime was effective enough (depicted on the left side of Figure 4) and whether they were willing to take additional medication to reduce the risk of this specific complication (depicted on the right side of Figure 4). Across all eight scenarios, the majority of respondents expressed a preference for taking additional medication to reduce the outlined risks. There appeared to be no coherence between the perception of whether the medication used was deemed sufficient and their willingness to take extra preventive medication during pregnancy. Even when most women deemed the medication sufficient, the preference for additional medication remained, as demonstrated in scenarios such as ‘hospitalisation during pregnancy’, ‘intrauterine growth restriction with delivery around the due date’, and ‘spontaneously preterm birth (28-37 weeks)’. In the scenario involving ‘intrauterine growth restriction with delivery round the due date’, 38% of respondents would refuse additional medication to prevent this outcome, representing the largest proportion declining.

When examining complications specific to the autoimmune diagnosis, all women diagnosed with APS expressed a desire for additional medication to prevent thrombosis. Among those diagnosed with SLE, 80% indicated a willingness to take additional medication to reduce the risk of neonatal lupus resulting in a heart block requiring pacemaker implantation, 57% would do so for the risk of neonatal lupus without a heart block and 76% would take additional medication to reduce the risk of SLE flares.

### Acceptability of side effects related to the use of biologic drugs

**Figure 5.**
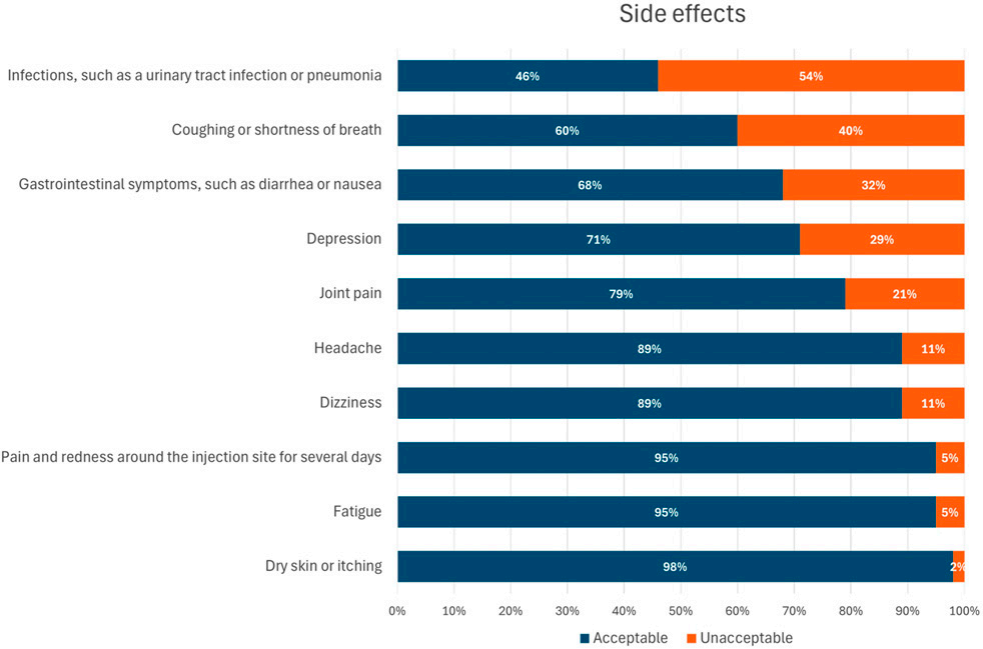
Acceptance rate of side effects associated with additional medication.

Respondents were questioned which side effects would refrain them from taking additional medication to reduce the risk of certain pregnancy complications. Respondents could answer either yes (it would refrain them) or no (it would not refrain them) and results are depicted in Figure 5. Most side effects associated with the use of biological disease modifying antirheumatic drugs were deemed acceptable by most respondents. However, a slight majority indicated they would refrain from additional medication if it led to an increased risk of infections such as a urinary tract infection or pneumonia. Other side effects that could discourage women from taking the medication included coughing or shortness of breath, gastrointestinal symptoms such as diarrhea or nausea, depression, and joint pains.

### Analysis of the open-ended questions

The questionnaire comprised three open questions: 1) ‘*What constitutes a successful pregnancy, and how can healthcare contribute to achieve this?*’, 2) ‘*What defines an unsuccessful pregnancy, and how can it be improved?’,* and 3) ‘*What would you like to share with us about your pregnancy experiences?*’. Response rates to these questions varied, with 52 (82%), 38 (60%), and 44 (70%) answers, respectively.

#### Successful pregnancy

A healthy child, and a mother who is able to take care of it!

Parents should feel heard, and their complaints should be taken seriously.

In 31 out of 52 responses (60%), the primary focus for a successful pregnancy was identified as the delivery of a healthy baby. Moreover, 14 (27%) respondents emphasized the importance of maternal health and the ability to nurture the newborn. However, the exact contribution of the healthcare system in attaining these goals remained unclear. While two women expressed a desire for more frequent check-ups, another respondent preferred minimal interference (*n* = 1). Nevertheless, the prevailing sentiment underscored the importance of personalized care, prioritizing individual needs over standardized healthcare interventions, as articulated by six women.

#### Unsuccessful pregnancy

The impossible sorrow of not becoming a mother.

Thirty-seven percent (14/38 respondents) stated that an unsuccessful pregnancy is defined as being unable to conceive or experiencing (recurrent) miscarriages. Forty-two percent labelled an unsuccessful pregnancy as a pregnancy resulting in stillbirth or resulting in the birth of a baby with negative health impacts. Adverse maternal health impacts or a prolonged recovery after pregnancy were labelled as an unsuccessful pregnancy by 29% of respondents. Fear and anxiety surrounding the pregnancy or their medical diagnosis were mentioned by five respondents.

#### Pregnancy experiences

Due to my diagnosis, I fear becoming pregnant.

‘I’ve experienced multiple miscarriages and a highly complicated pregnancy, but thankfully, I now have a healthy child’.

After enduring much pain and sorrow, we ultimately gave up hope.

**Figure 6.**
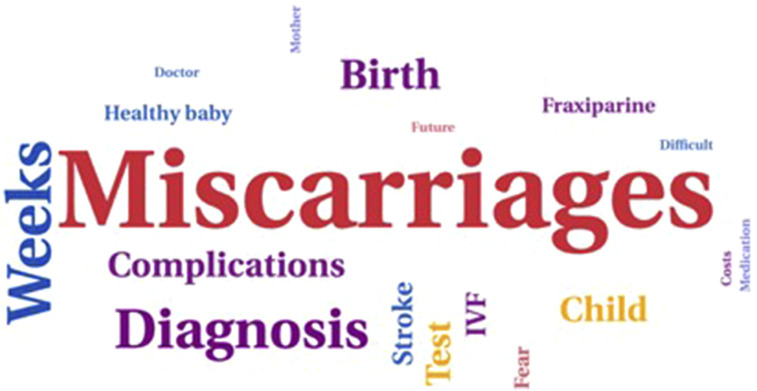
Most frequent words used in response to the question about pregnancy experiences.

The responses to this question were rich with emotions. Figure 6 shows the most frequent used words in the answers to this question, where the size of each word corresponds to its frequency of use. Some women faced challenges in conceiving due to subfertility or strong discouragement to become pregnant. Others feared pregnancy because of their medical diagnoses. Women who had experienced one or multiple pregnancies recounted numerous complications and miscarriages. Some women received their diagnosis only after experiencing multiple miscarriages. Some respondents expressed that they felt that the healthcare system fell short in providing adequate support. Many felt neglected and misunderstood by their doctors. They perceived a lack of individualized care, with medical protocols being prioritized over their personal circumstances, leading to significant medical oversights.

## Discussion

This study investigated patients’ perspectives on pregnancy complications in women with SLE and/or APS and studied their willingness to use additional medication during pregnancy to reduce the risk of adverse events. The study identified recurrent pregnancy loss, extreme preterm birth and severe pre-eclampsia as the three most critical complications to avoid. The results suggest that most patients prefer additional medication to reduce the risk of complications during pregnancy and show a generally high acceptance rate for side effects associated with the use of biological disease modifying anti-rheumatic drugs. With that, the results of this study give a voice to women with SLE and/or APS in the direction of future research on pregnancy management and outcomes. Lastly, the results highlight the significant mental burden of pregnancy complications in women with SLE and/or APS, underlining the need for improved treatment strategies and enhanced mental support during their reproductive years.

Previous studies on reproduction in patients with SLE and/or APS primarily focused on patients’ considerations regarding family planning^13,14^ and preferences regarding pre-pregnancy counselling.^15^ These studies, however, do not explore patients’ preferences and desires for medication use during pregnancy.

Overall, the results of this study suggest a preference for additional medication among participants to prevent complications during pregnancy. The willingness to consider additional medication was assessed across various situations and presented in different formats. In the vignettes describing mild pre-eclampsia, severe pre-eclampsia, and recurrent pregnancy loss, the desire for additional medication to lower the risk of these complications occurring ranged from 61% to 84%. Also, across eight briefly described scenarios or pregnancy complications as depicted in Figure 4, this desire for additional therapy to lower the risk of complications ranged from 51% to 89%. It is worth noting that the level of prior knowledge in our study population was unclear, and we did not, for example, explain for example that mild pregnancy complications could lead to lifelong consequences for the unborn baby. If participant had been better informed, their desire for medication might have been even higher. Additionally, there appeared to be no association between whether women perceive an adverse event as a complication of their rheumatic disease during pregnancy and their desire for new preventive medication. This unconditional desire for additional medication seems to contradict the findings of studies investigating the perceptions of women with inflammatory bowel disease regarding their disease, medication use, and pregnancy. These studies suggest that women often choose to endure active inflammatory disease rather than to use disease-modifying drugs because of concerns about medication safety during pregnancy and lactation.^16–20^ This discrepancy could possibly be explained by the well-known association between active disease and adverse pregnancy outcomes in patients with SLE, which could make them more receptive to medication use during pregnancy.

The analysis of open-ended questions highlighted a strong desire for personalized care among patients with SLE and/or APS during pregnancy. This finding was consistently referenced throughout the analysis. This study was not the first to highlight this need; similar findings have been reported in previous research involving patients with SLE, APS, inflammatory bowel disease, and rare connective tissue disorders.^14,21–23^ Indeed, research indicates that physicians face obstacles and complexities when engaging with women of reproductive age who have chronic conditions regarding their treatment and disease management before and during pregnancy.^15,24,25^ This emphasized the need for multidisciplinary clinical pathways, including mental support, throughout the complete trajectory starting at preconception counselling till the puerperium.^15^

The results of this study are limited by the relatively small sample size and response rate of <10%, which may not accurately represent the general population of SLE and/or APS and patients and could therefore bias study results. For instance, women who suffered multiple pregnancy complications might be more eager to participate in the online questionnaire as compared to women with SLE and/or APS who had more favourable pregnancy outcomes. Such a selection bias could potentially lead to strong opinions regarding medication use or the health care system. For this current project, patients were invited via the patient association NVLE, using social media and a webinar to response to the questionnaire. This unfortunately yielded an unanticipated low response rate. For future research, we will aim to improve engagement by collaborating with national rheumatology and obstetric societies. These societies could encourage rheumatologists and gynecologists to promote the questionnaire, for example, by distributing a leaflet with a QR code linking directly to the survey. This approach would not only potentially boost participation but also reach patients who may not be connected with the patient association, thus enhancing the representativeness of the sample. Additionally, because of the lack of a validated questionnaire, a new questionnaire was created. This makes it challenging to compare the results of this study to other studies. Additionally, while we aimed to explain pregnancy complications in layman’s terms, participants who have not experienced these issues firsthand may struggle to fully grasp the impact of the described adverse events. This could have influenced the responses to the questionnaire, such as the fact that 25% of patients were willing to accept thrombosis as a pregnancy complication if pregnancy would result in a live-born neonate.

This questionnaire marked the initial phase in uncovering unmet needs concerning pregnancy, pregnancy complications, and medication use during pregnancy among patients with SLE or APS. While some conclusions can be drawn, there are numerous avenues for further research. Future research should further explore these perspectives in larger-scale studies, aiming for a higher response rate to overcome the limitations associated with small sample sizes and potential selection bias of our study. Second our study did not explore the beliefs or expectations regarding the use of additional medication and its perceived risk of pregnancy complications. Gaining insight into the underlying factors driving these preferences can provide valuable insights for healthcare providers in tailoring treatment plans to meet patients’ needs. To gain these insights, it could be useful to use other research methods, such as a focus group, proving more detailed information.

In conclusion, we assessed the perspectives of women with SLE and/or APS on pregnancy, pregnancy complications, and medication use during pregnancy. We found that most women would accept complications throughout gestation if it results in a healthy baby. Also, a consistent majority of respondents expressed a desire for additional medication to reduce the risk of pregnancy complications, regardless of the specific complication at hand. Future research should delve deeper into the underlying reasons driving patients’ preferences for additional medication.

## Supplemental material

Supplemental Material - Patient perspectives on SLE, refractory APS, and biologic drug use during pregnancy Supplemental Material for Patient perspectives on SLE, refractory APS, and biologic drug use during pregnancy by Jara van Woerkom, Merlijn Wind, Yoe Kie Onno Teng, Titia Lely, Jamy Pullen, Gerard Jansen, Luis Perez-Garcia, Radboud Dolhain, Maarten Limper, Judith Kooiman in Lupus
