# Supplementary material for: Patient perspectives on SLE, refractory APS, and biologic drug use during pregnancy
Source: Lupus. 2026 Mar 26;35(7):699–708. doi: 10.1177/09612033261437466 (PMC13129208; doi:10.1177/09612033261437466)
Supplement: Supplemental Material - Patient perspectives on SLE, refractory APS, and biologic drug use during pregnancy [file sj-pdf-1-lup-10.1177_09612033261437466.pdf]

Appendix I – comprehensive search strategy

The search terms were based on the following DDO:

- *Domain*: Patients with SLE and/or APS*
- *Determinant*: Questionnaires
- *Outcome*: Patients views about medication use during pregnancy

("Questionnair*"[Title/Abstract] OR "Surveys and Questionnaires"[Mesh] OR "Survey*"[Title/Abstract] OR "Poll*"[Title/Abstract] OR "Inquir*"[Title/Abstract])
AND
("Patient*"[Title/Abstract] OR "Patients"[Mesh] OR "Individual*"[Title/Abstract] OR "Participant*"[Title/Abstract])
AND
("Autoimmune disease*"[Title/Abstract] OR "Autoimmune Diseases"[Mesh] OR "Chronic disease*"[Title/Abstract] OR "Chronic Disease"[Mesh] OR "Chronic Illness*"[Title/Abstract] OR "Chronic Condition*"[Title/Abstract] OR "Disease"[Mesh] OR "Disease*"[Title/Abstract] OR “Rheum*”[Title/Abstract] OR "Rheumatic Diseases"[Mesh])
AND
("Opinion*"[Title/Abstract] OR "Public Opinion"[Mesh] OR "Attitude"[Mesh] OR "Attitude*"[Title/Abstract] OR "Perception*"[Title/Abstract] OR "View*"[Title/Abstract] OR “Perspect*”[Title/Abstract])
AND
("Medication*"[Title/Abstract] OR "Drug*"[Title/Abstract] OR "Pharmaceutical*"[Title/Abstract] OR "Pharmaceutical Preparations"[Mesh] OR "Medicine*"[Title/Abstract] OR “Therap*”[Title/Abstract])
AND
("Pregnanc*"[Title/Abstract] OR "Pregnancy"[Mesh] OR "Gestation"[Title/Abstract] OR “Antenatal*”[Title/Abstract] OR “Obstetric*”[Title/Abstract])

472 results

* To broaden our search, we did not solely focus on patients with SLE or APS.

Appendix II – questionnaire

Dear members of the NVLE,

Thank you very much for your interest and participation in this survey. My name is Judith Kooiman, and I am a resident in the final stages of my gynecology training. In my future work as a gynecologist, I aim to focus on pregnant women with rheumatic diseases. Why this specific group? Because I am deeply concerned that pregnancies in this group can sometimes be

complicated, and I believe there is room for improvement. Scientific research is needed to improve health outcomes, and for that, I am asking for your help through this survey.

Women with APS (Antiphospholipid Syndrome) are typically treated with aspirin (Ascal) and blood thinners (low-molecular-weight heparin) during pregnancy. Patients with SLE (Systemic Lupus Erythematosus) often take aspirin and Plaquenil (hydroxychloroquine) in addition to their standard medications. These treatments are intended to reduce the risk of complications such as miscarriage, pregnancy-induced hypertension, or restricted fetal growth. However, these issues still occur more frequently in women with SLE or APS compared to the general population. I would like to hear from you about which pregnancy-related problems should be minimized by medication. And is preventing these problems worth the use of additional medication? By answering the questions in this survey, you will help us understand which women with SLE or APS might require new medications during pregnancy.

Filling out the questionnaire will take approximately 10 minutes. The results will be used anonymously. We understand that this survey may address a sensitive topic. We are very grateful for your responses.

Kind regards, on behalf of the entire research team,

Dr. Judith Kooiman, resident in gynecology and project leader

Jamy Scheerhoorn-Pullen, patient representative NVLE

►General questions

1. What is your age? *

What is your received diagnosis (multiple answers are possible)

- ○ APS (Antiphospholipid Syndrome)
- ○ SLE (Systemic Lupus Erythematosus)
- ○ Other rheumatic disease, specifically:

2. Do you currently, or did you at one point, have a desire to have children? *
   - ○ Yes
   - ○ No

3. If yes, has this desire to have children resulted in a live birth? (multiple answers possible)
   - ○ Yes
   - ○ No

4. What would you like to share with us about your pregnancy experiences?

5. According to you, when has a pregnancy been successful? How can healthcare make a difference in this regard?

6. When do you consider a pregnancy not to have been successful? What could or should be improved?

*Compulsory question

► Complications during pregnancy

7. What complications would you accept if your baby is born healthy? (multiple answers possible)
   - ○ SLE flare-up[1]
   - ○ Pre-eclampsia / HELLP syndrome[2]
   - ○ Thrombosis[3]
   - ○ None of the above

1. SLE flare-up = increase in SLE symptoms or abnormalities in blood tests, typically requiring additional medication.

2. Pre-eclampsia and HELLP syndrome = preeclampsia involves high blood pressure combined protein loss from the kidneys or growth restriction of the fetus. Patients may experience headaches, pain in the upper abdominal area, fluid retention, or nausea. Preeclampsia can be dangerous for the pregnant woman and may lead to seizures. Hospitalization is often necessary, and medications are administered to reduce the blood pressure. The condition only resolves after delivery. Therefore, delivery is sometimes induced before the due date.

3. Thrombosis = thrombosis occurs when a blood vessel becomes blocked by a blood clot. If the clod dislodges, it can end up in other organs (the lungs for example), leading to temporary or permanent impairment. Some patients may die as a result for thrombosis.

Below, three vignettes are described. Fir each case, three questions are provided.

►Vignette 1

A woman develops mild preeclampsia (high blood pressure and symptoms such as headaches, fluid retention and pain in the upper abdomen) despite standard therapy. Her baby is born healthy around the due date.

►Background information: Preeclampsia occurs in approximately 5% of pregnancies in the general population. In women with APS, this percentage is around 15%, and in women with SL, it's approximately 13%. Most cases of preeclampsia in women involve the mild variant that develops towards the end of pregnancy.

8. Do you consider mild preeclampsia to be a pregnancy complication?
    - ○ Yes
    - ○ No

9. Do you think the medication has been effective enough?
    - ○ Yes
    - ○ No

10. Would you be willing to take additional medication to reduce the risk of mild preeclampsia?
    - ○ No (no additional medication)
    - ○ Probably not
    - ○ I don't know

- Probably yes
- Yes (yes, additional medication)

►Vignette 2

A woman develops severe preeclampsia at 32 weeks of gestation, resulting in her baby being born prematurely.

11. Do you consider mild preeclampsia to be a pregnancy complication?
    - Yes
    - No

12. Do you think the medication has been effective enough?
    - Yes
    - No

13. Would you be willing to take additional medication to reduce the risk of mild preeclampsia?
    - No (no additional medication)
    - Probably not
    - I don’t know
    - Probably yes
    - Yes (yes, additional medication)

►Vignette 3

A woman has multiple miscarriages. After several years, she has a successful pregnancy and brings home a healthy baby.

►Background information: Recurrent miscarriages (2 or more) occur in approximately 1-2% of women in the general population. In women with APS, this percentage is around 52%, and in women with SLE, it's approximately 11%.

14. Do you consider mild preeclampsia to be a pregnancy complication?

- ○ Yes
- ○ No

15. Do you think the medication has been effective enough?

- ○ Yes
- ○ No

16. Would you be willing to take additional medication to reduce the risk of mild preeclampsia?

- ○ No (no additional medication)
- ○ Probably not
- ○ I don't know
- ○ Probably yes
- ○ Yes (yes, additional medication)

►Do you think the medication has worked well enough if the following issues arise during pregnancy?

17. Please select the most appropriate answer

	The medication has worked sufficiently	The medication has not worked sufficiently

Spontaneous premature birth (28 – 37 weeks)	O	O
Spontaneous extreme premature birth (24 – 28 weeks). At this gestational age, there is a higher risk of death or lasting damage to the baby.	O	O
Pregnancy loss (before 24 weeks). These babies do not survive.	O	O
Recurrent miscarriages and ultimately, no liveborn baby.	O	O
Intrauterine growth restriction with delivery around the due date. These babies may require extra care after birth but generally can go home quickly.	O	O
Intrauterine growth restriction with preterm delivery. These babies are admitted to the intensive care unit.	O	O
Hospital admission during pregnancy.	O	O
Admission of the baby to the intensive care unit or other department of the hospital.	O	O

18. Would you be willing to take additional medication to reduce the risk of the following issues during pregnancy?

	No (no additional medication)	Probably not	I don't know	Probably yes	Yes (additional medication)
Spontaneous premature birth (28 – 37 weeks)	O	O	O	O	O
Spontaneous extreme premature birth (24 – 28 weeks). At this gestational age, there is a higher risk of death or lasting damage to the baby.	O	O	O	O	O
Pregnancy loss (before 24 weeks). These babies do not survive.	O	O	O	O	O
Recurrent miscarriages and ultimately, no liveborn baby.	O	O	O	O	O
Intrauterine growth restriction with delivery around the due date. These babies may require extra care after birth but generally can go home quickly.	O	O	O	O	O
Hospital admission during pregnancy.	O	O	O	O	O
Admission of the baby to the intensive care unit or other department of the hospital.	O	O	O	O	O

19. Do you have the diagnosis of APS? *

- ○ Yes
- ○ No

20. Would you be willing to take additional medication to prevent thrombosis during pregnancy?

	No (no additional medication)	Probably not	I don't know	Probably yes	Yes (additional medication)
Thrombosis	O	O	O	O	O

21. Do you have the diagnosis of SLE?

- ○ Yes
- ○ No

22. Would you be willing to take additional medication to prevent the following issues?

	No (no additional medication)	Probably not	I don't know	Probably yes	Yes (additional medication)
SLE flare-up	O	O	O	O	O
Temporary lupus symptoms in the baby[4]	O	O	O	O	O
Implantation of a pacemaker in the baby[4]	O	O	O	O	O

4. Lupus symptoms in the baby = Neonatal lupus is a rare condition in newborn babies, caused by SLE antibodies from the mother reaching the baby through the placenta. In most cases, the symptoms of neonatal lupus disappear spontaneously after a few months. The most common symptom is a rash.
In the most severe cases, congenital heart block can occur, where the baby has a slow heart rate. Since this condition does not resolve on its own, these babies receive a pacemaker (a device placed under the skin to regulate the heart rate) after birth.

* Compulsory question

►Side effects

23. Additional medication carries the risk of side effects. What side effects could make you unwilling to take medication, even if the medication reduces the risk of pregnancy complications? (multiple answers possible)
    - ○ Pain and redness around the injection site for several days
    - ○ Dry skin or itching
    - ○ Infections, such as urinary tract infection or pneumonia
    - ○ Gastrointestinal symptoms such as diarrhea or nausea
    - ○ Coughing or shortness of breath
    - ○ Joint pains
    - ○ Dizziness
    - ○ Head ache
    - ○ Fatigue
    - ○ Depression
    - ○ Other

Thank you for your participation
